# Prognostication of Liver Disease Patients via the WFUMB ‘Rule‐of‐4’ Algorithm Using SSI‐2D‐SWE‐Based Liver Stiffness Measurement

**DOI:** 10.1111/liv.70614

**Published:** 2026-04-09

**Authors:** Christian Sebesta, Georg Kramer, Nina Dominik, Benedikt S. Hofer, Lorenz Balcar, Paul Thöne, Mathias Jachs, Lukas Hartl, Benedikt Simbrunner, Till Schöchtner, Friedrich Haimberger, Nicolas Balutsch, Albert F. Stättermayer, Michael Trauner, Mattias Mandorfer, Thomas Reiberger, David J. M. Bauer

**Affiliations:** ^1^ Division of Gastroenterology and Hepatology, Department of Internal Medicine III Medical University of Vienna Vienna Austria; ^2^ Vienna Hepatic Hemodynamic Lab, Division of Gastroenterology and Hepatology, Department of Internal Medicine III Medical University of Vienna Vienna Austria; ^3^ LBG Clinical Research Group MOTION Medical University of Vienna Vienna Austria; ^4^ Christian‐Doppler Laboratory for Portal Hypertension and Liver Fibrosis Medical University of Vienna Vienna Austria; ^5^ Department of Internal Medicine IV Klinik Ottakring Vienna Austria

## Abstract

**Background:**

The World Federation for Ultrasound in Medicine and Biology (WFUMB) guidance update 2024 proposed a ‘rule‐of‐4’ using ARFI liver stiffness measurement (LSM) to stratify the risk of decompensation events. This rule identifies advanced chronic liver disease (ACLD) at a threshold of ≥ 13 kPa and indicates a high probability of clinically significant portal hypertension (CSPH) above 21 kPa.

**Methods:**

We prospectively enrolled 187 patients undergoing same‐day SSI‐2D‐LSM (Aixplorer SuperSonic Imagine) and hepatic venous pressure gradient (HVPG) measurement. Patients were stratified into three LSM groups < 13 kPa, 13–21 kPa and > 21 kPa and followed for decompensation events for 1 year (Y1).

**Results:**

The cohort comprised 31 (16.6%) patients with LSM < 13 kPa (non‐ACLD); 33 (17.6%) LSM 13–21 kPa (ACLD) and 123 (65.8%) LSM > 21 kPa (high CSPH probability). The corresponding median HVPG values were 5 [IQR: 3–7] vs. 10 [7–14] vs. 17 [13–21] mmHg, respectively, and the corresponding CSPH prevalence was 6.5%, 54.5% and 91.9%. In ROC analysis to predict CSPH, SSI‐2D‐LSM demonstrated high accuracy (AUC: 0.83). Validating the new WFUMB extension of the ‘rule‐of‐4’, the recommended > 21 kPa threshold identified CSPH with a specificity of 80.0% and a PPV of 83.1%. In competing risk analysis, the Y1‐decompensation rate was 0%, 10% and 24.7% of patients in the < 13 kPa, 13–21 kPa and > 21 kPa groups, respectively. For prediction of Y1‐decompensation risk in the compensated ACLD (cACLD) subgroup, HVPG demonstrated the highest predictive accuracy (AUC: 0.92), while SSI‐2D‐LSM (AUC: 0.75) performed similarly to vibration‐controlled transient elastography (VCTE: AUC: 0.77; AUC‐comparison: *p* = 0.837).

**Conclusions:**

The WFUMB ‘rule‐of‐4’ for ARFI‐LSM allows for accurate and point‐of‐care non‐invasive risk stratification of patients with liver disease. Specifically, the short‐term risk of decompensation starts at SSI‐2D‐LSM ≥ 13 kPa and becomes considerable at SSI‐2D‐LSM > 21 kPa (indicating high CSPH probability). These findings support the broader implementation of ARFI‐LSM for risk assessment in clinical routine.

## Summary

Advanced liver fibrosis and portal hypertension can lead to serious complications, but assessing this risk typically requires invasive liver vein pressure gradient measurements. This study shows that an ultrasound‐based liver stiffness test (ARFI‐LSM) can reliably identify patients at low, moderate, or high risk of liver decompensation. Using two key thresholds (13 kPa and 21 kPa), clinicians can quickly estimate risk at bedside and decide who needs closer monitoring or treatment.

## 1 Introduction

Clinically significant portal hypertension (CSPH) is a key determinant of decompensation risk in patients with advanced chronic liver disease (ACLD) [1, 2, 3]. Decompensation is defined by the development of ascites, variceal bleeding, or hepatic encephalopathy, significantly worsening prognosis. While the hepatic venous pressure gradient (HVPG) remains the gold standard for diagnosing CSPH, its widespread use is limited by the need for specialised expertise and equipment, rendering it inaccessible in many centers [4, 5].

This has propelled the development of non‐invasive liver stiffness measurement (LSM) techniques. Methods such as vibration‐controlled transient elastography (VCTE) and acoustic radiation force impulse (ARFI) offer practical, point‐of‐care alternatives for assessing CSPH risk [5, 6, 7, 8].

ARFI‐imaging is an ultrasound technique that tracks the movement of generated shear waves using B‐mode imaging, enabling the assessment of tissue elasticity [9]. There are two prominent ARFI techniques: point‐Shear Wave Elastography (pSWE) [10] and 2D‐SWE [11, 12, 13].

A key advantage of ARFI‐Imaging is its integration in modern ultrasound systems, allowing clinicians to screen for fibrosis, hepatocellular carcinoma (HCC), portal vein thrombosis (PVT) or other signs of ACLD during a single examination [14, 15, 16, 17].

ARFI is also feasible in patients with ascites, a clinical setting where VCTE is technically limited [18, 19]. Like VCTE, in addition to assessing liver fibrosis and PH [20, 21] ARFI also reflects inflammation, a factor in disease progression [22, 23, 24, 25]. Therefore, ARFI‐LSM represents a valuable prognostic tool in both compensated and decompensated liver disease [9, 22, 26, 27, 28].

To standardise its clinical application, the World Federation for Ultrasound in Medicine and Biology (WFUMB) recently proposed a ‘rule‐of‐4’ for ARFI‐LSM. This framework categorises LSM into different thresholds: an LSM ≤ 5 kPa suggests a normal liver, a value < 9 kPa can effectively rule out compensated ACLD (cACLD) and the 9–13 kPa range requires further evaluation. An LSM > 13 kPa confirms cACLD, > 17 kPa suggests the presence of CSPH and > 21 kPa indicates a high probability of CSPH. However, WFUMB underscored the critical need for prospective validation to confirm the rule's prognostic reliability [29]. Therefore, the primary aim of this study was to validate the prognostic accuracy of the ‘rule‐of‐4’ thresholds for predicting one‐year liver‐related events in a prospectively followed, HVPG‐characterised cohort.

## 2 Patients and Methods

### 2.1 Study Cohort

This prospective study enrolled patients at the Medical University of Vienna between July 2020 and August 2023. The cohort consisted of adults scheduled for a clinically indicated HVPG measurement who had also undergone same‐day Supersonic Imagine 2D‐Shearwave Elastography (SSI‐2D‐LSM) and laboratory testing. All included patients had a minimum follow‐up of 28 days.

**FIGURE 1:**
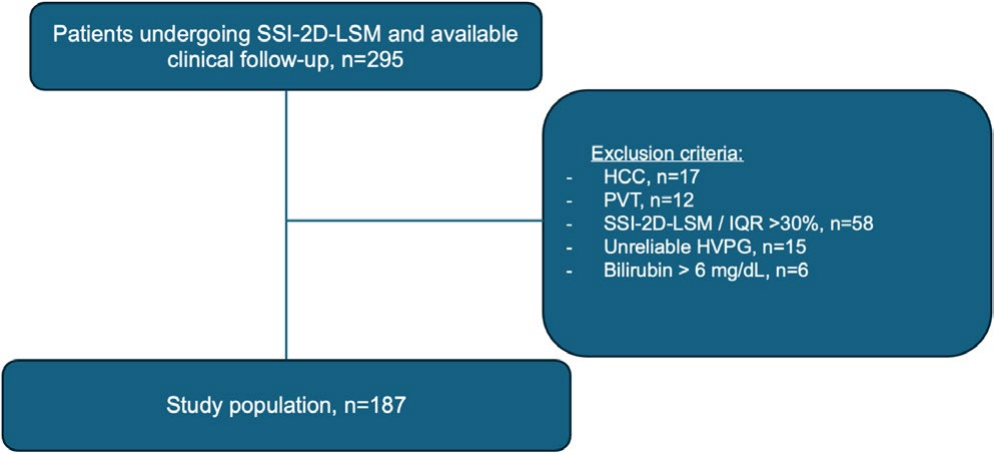
Patient flow chart. ALT, alanine transaminase; HCC, hepatocellular carcinoma; HVPG, hepatic venous pressure gradient; IQR, interquartile range; LSM, liver stiffness measurement; n, number; PVT, portal vein thrombosis; SSI, SuperSonic Imagine.

Exclusion criteria were hepatocellular carcinoma (HCC), prior transjugular intrahepatic portosystemic shunt (TIPS) or liver transplantation (LT), portal vein thrombosis (PVT), congestive heart failure or portosinusoidal vascular disease (PSVD), diagnosed via clinical and/or histological findings [30]. We also excluded patients with severe laboratory abnormalities (ALT > 250 U/L or total bilirubin > 6 mg/dL), technical failures or unreliable SSI‐2D‐LSM (IQR/median ratio > 30% [29]) or inadequate HVPG recordings. The patient selection process is detailed in Figure 1.

Patients were stratified into three analytical groups based on baseline SSI‐2D‐LSM values: < 13 kPa, 13–21 kPa and > 21 kPa. Decompensation was defined by Baveno VII criteria [1]. The study protocol was approved by the local ethics committee (EK‐number: 1544/2019) and all participants provided written informed consent. The study was conducted in accordance with the ethical principles of the Declaration of Helsinki and its 2024 revision.

### 2.2 Elastography

Patients fasted for at least 5 h before all elastography procedures. We performed all LSM examinations before the HVPG procedure to ensure operators were blinded to hemodynamic results. A team of 10 experienced operators (B.A.D., B.A.L., H.A.L., J.A.M., M.A.M., P.A.R., R.E.T., S.C.B., S.C.M., S.T.A.), each with a history of performing over 500 LSM procedures, conducted the examinations. For both techniques (VCTE, SSI‐2D‐SWE), patients were positioned supine with the right arm maximally abducted to widen the intercostal space. All measurements were taken in the mid‐axillary line.

ARFI‐LSM was performed using SuperSonic Imagine (SSI) 2D‐SWE on an Aixplorer Ultimate ultrasound platform (SuperSonic Imagine, Aix‐en‐Provence, France). With B‐mode imaging, we identified a target measurement area at least 2 cm deep to the liver capsule, ensuring it was free of large vessels or bile ducts. We instructed patients to hold their breath at mid‐exhalation during image acquisition. Within the target area, we acquired at least six valid measurements from two separate 1‐cm regions of interest (ROIs).

VCTE was performed with a FibroScan device (Echosens, Paris, France) according to international guidelines [1, 18]. In contrast to SWE, this technique does not require a patient breath‐hold or manual selection of a region of interest. The M‐probe was used by default, with the XL‐probe substituted when indicated by the device's automatic probe selection tool [31]. Ten valid acquisitions were obtained; results were expressed as the median liver stiffness value, with the IQR/Median as the quality criterion.

### 2.3 Laboratory and Clinical Data

We analyzed all blood samples at the ISO 15189‐certified central laboratory of the Medical University of Vienna/AKH Wien. We collected other clinical data, including comorbidities and prior diagnoses, from electronic patient records.

### 2.4 Statistical Analysis

We reported categorical variables as absolute numbers (N) and percentages (%) and compared them between groups using the Chi‐square test. We assessed continuous variables for normal distribution using the Shapiro–Wilk test; normally distributed data are presented as mean ± standard deviation (SD), while non‐normally distributed data are presented as median with interquartile range (IQR). We used the Kruskal‐Wallis test for between‐group comparisons of continuous variables.

To evaluate the ability of SSI‐2D‐LSM to identify specific outcomes, we used Receiver Operating Characteristic (ROC) curve analysis. For the SSI‐2D‐LSM threshold of > 21 kPa, as defined by the WFUMB ‘rule‐of‐4’ [29], we calculated sensitivity, specificity, positive and negative predictive values (PPV/NPV) and the positive diagnostic likelihood ratio (DLR+) for diagnosing CSPH. We compared the Areas Under the Curve (AUCs) of different elastography methods using the DeLong test.

To assess prognosis, we performed a competing risk analysis to model the one‐year risk of hepatic decompensation across the three SSI‐2D‐LSM subgroups. LT and non‐liver related mortality were treated as competing events. We also conducted a pre‐planned subgroup analysis comparing the predictive performance of the tests in patients with cACLD versus decompensated ACLD (dACLD).

All analyses were performed using R statistical software (version 4.3.2). A list of the specific R packages used is provided in the Supporting Information. The language and clarity of this manuscript were refined using the large language model Gemini 2.5 Pro (Google AI). The model's function was strictly confined to improving grammar, syntax and stylistic consistency. The AI did not contribute to the generation of scientific hypotheses, data analysis, interpretation of results, or the drawing of conclusions. All AI‐generated suggestions were critically reviewed by the authors, who retain full and final responsibility for all content within this manuscript.

## 3 Results

### 3.1 Patient Characteristics

Of 295 initially screened patients, 187 patients who did not meet the exclusion criteria were included in the final analysis (Figure 1; Table 1).

**TABLE 1:** Patient characteristics stratified by SSI‐2D‐LSM Groups.

	< 13 kPa	13–21 kPa	> 21 kPa	Overall	
	(*N* = 31)	(*N* = 33)	(*N* = 123)	(*N* = 187)	*p*
Age (years) [IQR]	55 [46–66]	56 [52–66]	56 [50–64]	56 [50–64]	0.940
Sex (F/M, %M)	10/21 (67.7%)	10/23 (69.7%)	39/84 (68.3%)	59/128 (64.8%)	0.984
BMI (kg/m2) [IQR]	25.39 [23.3–28.0]	24.3 [22.0–27.2]	26.2 [23.3–29.7]	25.7 [22.9–28.8]	0.290
Aetiology					
ALD (*n*, %)	3 (9.7%)	11 (33.3%)	78 (63.4%)	92 (49.2%)	**< 0.001**
MASLD (*n*, %)	2 (6.5%)	7 (21.2%)	10 (8.1%)	19 (10.2%)	
Viral (*n*, %)	9 (29.0%)	6 (18.2%)	16 (13.0%)	31 (16.6%)	
Other (*n*, %)	17 (54.8%)	9 (27.3%)	19 (15.4%)	45 (24.1%)	
VCTE LSM (kPa) [IQR]	9.7 [6.6–14.6]	17.1 [13.1–21.4]	48.3 [31.2–70.7]	30.9 [16.6–59.1]	**< 0.001**
Child Pugh Score (points) [IQR]	5 [5–5]	5 [5–6]	7 [5–8]	6 [5–8]	**< 0.001**
MELD (points) [IQR]	9 [7–11]	9 [7–10]	12 [10–16]	10 [9–15]	**< 0.001**
Platelet count (G/L) [IQR]	181 [135–205]	102 [83–165]	109 [69–153]	117 [77–171]	**< 0.001**
Thrombocytopenia < 150 G/L (*n*, %)	9 (29.0%)	22 (66.7%)	91 (74.0%)	122 (65.2%)	**< 0.001**
HVPG (mmHg) [IQR]	5 [3–7]	10 [7–14]	17 [13–21]	14 [9–20]	**< 0.001**
CSPH (*n*, %) [IQR]	2 (6.5%)	18 (54.5%)	113 (91.9%)	133 (71.1%)	**< 0.001**
Ascites (*n*, %)	0 (0%)	1 (3.0%)	23 (18.7%)	24 (12.8%)	**< 0.001**
Any varices (*n*, %)	1 (3.2%)	9 (27.3%)	68 (55.3%)	78 (41.7%)	**< 0.001**
High risk varices (*n*, %)	0 (0%)	2 (6.1%)	35 (28.5%)	37 (19.8%)	**< 0.001**
SSI‐2D‐LSM (kPa) [IQR]	10.0 [8.8–11.3]	15.9 [14.1–17.8]	58.4 [38.6–76.2]	37.2 [15.7–65.6]	**< 0.001**
Follow‐Up time (months) [IQR]	24.8 [12.5–27.9]	21.2 [13.5–26.0]	25.3 [13.1–28.5]	24.1 [13.1–28.3]	0.833
Decompensation rate at Y1 (*n*, %)	0 (0%)	3 (9.1%)	28 (22.8%)	31 (16.6%)	**0.012**

*Note: p*‐values comparing differences among groups < 0.05 bold.

Abbreviations: ALD, alcoholic liver disease; CSPH, clinically significant portal hypertension; HVPG, hepatic venous pressure gradient; IQR, interquartile range; LSM, liver stiffness measurement; M, Men; MASLD, metabolic associated steatotic liver disease; n, number; pts., points; SSM, spleen stiffness measurement; SWE, shear wave elastography; VCTE, vibration controlled transient elastography; W, Women; Y1, One year.

We stratified the cohort into three groups based on baseline SSI‐2D‐LSM values: < 13 kPa group (*n* = 31, 16.6%), 13–21 kPa group (*n* = 33, 17.6%) and > 21 kPa group (*n* = 123, 65.8%). Demographic characteristics were similar across the three strata (Table 1). There were no significant differences in median age (56 years [IQR: 50–64], *p* = 0.940) or BMI (25.7 kg/m^2^ [IQR: 22.9–28.8], *p* = 0.290). The overall cohort was predominantly male (64.8%). The underlying aetiology of liver disease differed significantly between the groups. The proportion of patients with alcoholic liver disease (ALD) rose with increasing stiffness, from 9.7% in the < 13 kPa group to 63.4% in the > 21 kPa group (*p* < 0.001). In contrast, metabolic dysfunction‐associated steatotic liver disease (MASLD) and viral hepatitis were more common in the lower SSI‐2D‐LSM categories (Table 1). Only six patients (3.7%) were receiving beta‐blocker therapy at the time of measurement.

Indicators of liver dysfunction worsened as SSI‐2D‐LSM values increased. Both Child‐Pugh and MELD scores rose progressively across the three groups (*p* < 0.001 for both), while platelet count (PLT) showed a significant inverse relationship (*p* < 0.001). Median HVPG increased significantly across the groups, from 5 mmHg (< 13 kPa) to 10 mmHg (13–21 kPa) to 17 mmHg (> 21 kPa) (*p* < 0.001). Consequently, the prevalence of CSPH rose from 6.5% in the lowest stiffness group to 91.9% in the highest (*p* < 0.001). Severe ascites (grade 3 [32]) was observed predominantly in the > 21 kPa group, affecting 18.7% of patients in this category. The median follow‐up period for the cohort was 24.1 months [IQR: 13.1–28.3] and did not differ significantly between the three SSI‐2D‐LSM groups (*p* = 0.833), ensuring a comparable observation period for outcomes (Table 1).

The baseline SSI‐2D‐LSM strata effectively stratified the one‐year risk for clinical decompensation (*p* = 0.012). The incidence of hepatic decompensation increased sharply with each stiffness category: the one‐year risk was 0% for patients in the < 13 kPa group, 9.1% for the 13–21 kPa group and 22.8% for the > 21 kPa group (Table 1).

To address the high prevalence of CSPH and the potential for spectrum bias, we characterised patients who underwent HVPG measurement but were not assessed via SSI‐2D‐LSM (*n* = 76). These patients were similar to those who underwent SSI‐2D‐LSM and HVPG (Table S1).

In 40 of 187 patients (21.5%) we obtained three measurements with a stability index (SI) ≥ 90%, the threshold recommended by the manufacturer [33], whereas 82 patients (43.9%) met a lower SI cutoff of ≥ 70%. However, the AUCs for CSPH for the SI ≥ 90% (vs. < 90%; *p* = 0.80) and ≥ 70% (vs. < 70%; *p* = 0.66) were not significantly improved when applying SI cut‐offs.

### 3.2 Analysis Using the Original Rule‐of‐Four Stratification (< 5, 5–9, 9–13, 13–17, 17–21, > 21)

In the subgroup analysis of the original rule‐of‐four groups (< 5, 5–9, 9–13, 13–17, 17–21, > 21 kPa; Table S2), baseline disease severity worsened stepwise with higher groups: VCTE‐LSM, Child‐Pugh score, MELD, HVPG, presence of CSPH, ascites and varices were all significantly (and gradually) higher in advanced groups (all *p* < 0.001) while PLT declined progressively (*p* < 0.001). Follow‐up duration was comparable across groups (*p* = 0.579). At 1 year, decompensation events occurred most frequently in the > 21 kPa group (22.8%), while no events were observed in the ≤ 13 kPa groups. Intermediate event rates were seen in the 13–17 kPa (9.5%) and 17–21 kPa (8.3%) groups (*p* = 0.053) (Table S2).

### 3.3 Predicting CSPH

**FIGURE 2:**
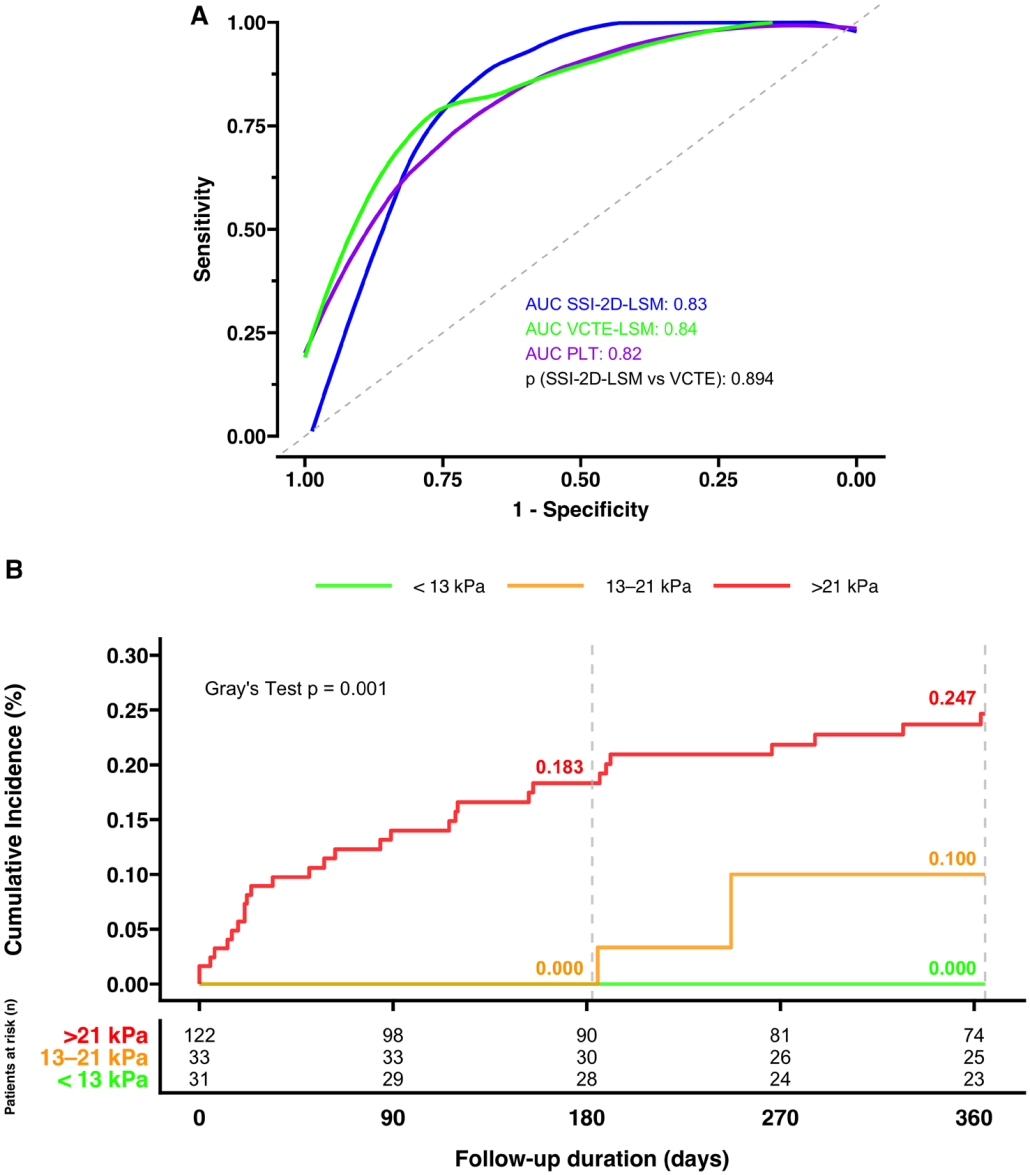
(A) ROC curve for CSPH prediction in cACLD patients. Comparison between the groups was done using the De‐Longs Test. (B) Cumulative Incidence of hepatic decompensation stratified by SSI‐2D‐LSM – considering liver transplantation and non‐liver‐related death as competing risks. CSPH, clinically significant portal hypertension; LSM, liver stiffness measurement; n, number; PLT, platelets; SSI, Supersonic Imagine; VCTE, vibration controlled transient elastography.

In ROC analysis to predict CSPH in cACLD, SSI‐2D‐LSM demonstrated high accuracy (AUC = 0.83; Figure 2A). A threshold of SSI‐2D‐LSM > 21 kPa as suggested by the rule‐of‐4 identified CSPH with a sensitivity of 76.6%, a specificity of 80.0%, a PPV of 83.1% and a positive diagnostic likelihood ratio (DLR^+^) of 3.83 (Table 2).

**TABLE 2:** Diagnostic performance of SSI‐2D‐LSM thresholds for ruling in/ruling out CSPH.

Cutoff	Sensitivity	Specificity	PPV	NPV	DLR^+^	DLR^−^
< 9 kPa^a^	**100%**	14%	60%	**100%**	1.16	**0.00**
< 13 kPa	**97%**	54%	73%	**93%**	2.11	**0.06**
< 13 kPa & PLT ≥ 150 G/L	**100%**	42%	69%	**100%**	1.72	**0.00**
> 17 kPa	80%	**68%**	**76%**	72%	**2.49**	0.30
> 17 kPa & PLT < 150 G/L	66%	**78%**	**79%**	64%	**2.98**	0.44
> 21 kPa	77%	**80%**	**83%**	73%	**3.83**	0.20
> 21 kPa & PLT < 150 G/L	64%	**88%**	**87%**	66%	**5.34**	0.41

*Note:* Metrics relevant to rule‐in/rule‐out bold.

Abbreviations: CSPH, Clinically significant portal hypertension; DLR−, Negative diagnostic likelihood ratio; DLR+, Positive diagnostic likelihood ratio; LSM, Liver stiffness measurement; NPV, Negative predictive value; PLT, Platelets; PPV, Positive predictive value; SSI‐2D‐LSM, SuperSonic Imagine 2D shear‐wave liver stiffness measurement; SWE, Shear‐wave elastography.

^a^ Only 8 patients in the < 9 kPa group.

Combining SSI‐2D‐LSM platelets in analogy to the rule‐of‐five further improved rule‐in accuracy: LSM > 21 kPa + PLT < 150 G/L achieved a specificity of 88.0% and a PPV of 87.2% (DLR^+^ = 5.34), representing the best‐performing rule for confirming CSPH (Table 2).

For ruling out CSPH, an SSI‐2D‐LSM ≤ 13 kPa combined with PLT ≥ 150 G/L identified patients without CSPH with 100% sensitivity, 42% specificity and a NPV of 100%, corresponding to a DLR^−^ of 0.00 (Table 2).

### 3.4 Predicting Hepatic Decompensation Events: One‐Year Prognostic Assessment

In a subgroup analysis limited to patients with cACLD, HVPG demonstrated excellent prognostic accuracy for predicting one‐year decompensation (AUC: 0.92). The non‐invasive methods also performed well, with VCTE‐LSM showing an AUC of 0.77 and SSI‐2D‐LSM an AUC of 0.75. A pairwise comparison confirmed that the prognostic performance of the two elastography methods was not significantly different (*p* = 0.837).

When we expanded the analysis to the entire cohort, including patients with dACLD, the prognostic accuracy for predicting a first or subsequent decompensation event was more modest for all methods. HVPG remained the strongest predictor (AUC: 0.80). VCTE‐LSM (AUC: 0.69) and SSI‐2D‐LSM (AUC: 0.69) showed moderate performance, and again, the difference between them was not statistically significant (*p* = 0.92).

We then evaluated the clinical utility of the pre‐defined WFUMB threshold of SSI‐2D‐LSM > 21 kPa for one‐year prognosis in the overall cohort. The cutoff showed high sensitivity (90%) but low specificity (39%). This profile resulted in a low PPV of 23%. However, it yielded a high NPV of 95%, suggesting that a value below 21 kPa effectively rules out the short‐term risk of decompensation. The DLR+ was 1.48.

In the cACLD group, the cutoff demonstrated a high sensitivity of 85% for predicting one‐year decompensation, while specificity improved to 52%. This resulted in a slightly lower PPV of 19%, compared to the overall cohort, but yielded an even higher NPV of 96%. The DLR+ increased to 1.78.

### 3.5 Competing Risk Analysis: One‐Year Decompensation Assessment

To assess the cumulative incidence of hepatic decompensation, we performed a competing risk analysis accounting for LT and non‐liver‐related death as competing events.

Cumulative incidence estimates at six and 12 months demonstrated a clear risk gradient across the SSI‐2D‐SWE‐based liver stiffness strata. At 6 months, the cumulative incidence of decompensation events was 0% in patients with an LSM < 13 kPa, 0% in those with an LSM of 13–21 kPa and 18.3% in those with an LSM > 21 kPa. At 12 months, the corresponding incidences were 0%, 10.0% and 24.7%, respectively (Figure 2B). Grey's test confirmed a significant difference in cumulative incidence across the three liver stiffness categories (*p* = 0.001).

However, the absence of decompensation events in the < 13 kPa reference group precluded a reliable estimation of subdistribution hazard ratios. To address this limitation, we dichotomised the cohort at the clinically relevant threshold of 21 kPa (LSM ≤ 21 vs. > 21 kPa). This approach enabled a Cox proportional hazards model, which revealed that patients with an LSM > 21 kPa had a nearly five‐fold increased hazard for a decompensation event compared to those with an LSM ≤ 21 kPa (Hazard Ratio [HR] = 4.85, 95% Confidence Interval [CI]: 1.91–12.3, *p* = 0.001).

In a subgroup competing risk analysis including only patients with cACLD, the cumulative incidence of first decompensation at 6 months was 0% in both lower SSI‐2D‐LSM groups, while 8.7% of patients in the > 21 kPa group experienced decompensation. At 12 months, no decompensation events occurred in the < 13 kPa group, but 8.7% and 20.1% of patients in the 13–21 kPa and > 21 kPa groups, respectively, experienced decompensation. Grey's test confirmed a significant difference in cumulative incidence among groups (*p* = 0.006) (Figure S1).

Given to the absence of events in the < 13 kPa group, the cohort was subsequently dichotomised at 21 kPa. Cox regression analysis confirmed that patients with LSM > 21 kPa had a significantly increased risk compared with those SSI‐2D‐LSM ≤ 21 kPa (HR 4.38, 95% CI 1.47–13.0, *p* = 0.008).

## 4 Discussion

This study validates the clinical significance of the WFUMB ‘rule‐of‐4’ for ARFI‐based LSM [29] and confirms the prognostic value of the > 21 kPa threshold for identifying patients at high risk.

The increasing prevalence of CSPH across the WFUMB ‘rule‐of‐4’ categories underscores the relevance of these thresholds. In particular, the > 21 kPa cutoff effectively identified patients with CSPH, reinforcing its clinical utility. Our findings are consistent with those of Heilani et al. [34], who proposed 2D‐SWE‐LSM thresholds of > 18.5 kPa to rule in and < 10 kPa to rule out CSPH, while identifying a VCTE cutoff of > 25 kPa, in line with the Baveno consensus [1, 34]. Their study further showed that, among ARFI techniques, 2D‐SWE provided the best diagnostic performance, whereas pSWE achieved high sensitivity but limited specificity.

Previous ARFI data using Siemens Virtual Touch Tissue Quantification (a point SWE technique) [35] demonstrated a stepwise increase of liver and spleen stiffness with rising HVPG and a high accuracy for diagnosing CSPH (AUC up to 0.97) [35]. Another recent study using 2D‐SWE‐LSM from General Electric [36] likewise demonstrated an excellent performance for CSPH detection (AUC 0.91), which was non‐inferior to VCTE (AUC 0.92; *p* = 0.79) when applying a cutoff of 11.3 kPa [36].

Importantly, our study also shows that SSI‐2D‐LSM performs comparably to VCTE for CSPH prediction, with no significant difference in their diagnostic performance. Given that ARFI‐based techniques are integrated into standard ultrasound platforms, they represent an accessible and pragmatic tool for routine clinical use, allowing LSM alongside screening for HCC and other cirrhosis‐related features in a single examination [14, 15].

In addition to its accessibility, ARFI‐LSM holds other key advantages over VCTE‐LSM, including its proven feasibility in patients with ascites [14, 19]. Furthermore, LSM may reflect not only fibrosis and HVPG but also hepatic inflammation, a critical determinant of disease progression in ACLD [23, 24, 37, 38].

In line with current guidelines [1, 29, 33], our study provides a prospective validation of the WFUMB ‘rule‐of‐4’ using HVPG and complements the VCTE‐based Baveno VII thresholds, supporting 2D‐SSI‐LSM as a robust tool for non‐invasive CSPH prediction. This suggests that ARFI‐LSM could be a valuable tool for predicting both initial decompensation in cACLD and the risk of further decompensation in dACLD, thereby aiding clinical management across different stages of advanced liver disease.

Despite this potential, our results highlight important limitations. In our cohort, SSI‐2D‐LSM demonstrated only moderate overall accuracy for predicting a decompensation event within 1 year, performing similarly to VCTE‐LSM. While the WFUMB‐recommended cutoff of > 21 kPa yielded a high sensitivity for identifying at‐risk patients, its utility was diminished by a low specificity and a low PPV. This combination indicates a high false‐positive rate, as most patients with an LSM > 21 kPa did not experience a decompensation event during follow‐up. In contrast, the high NPV provides strong clinical reassurance: patients with an SSI‐2D‐LSM ≤ 21 kPa have a very low likelihood of near‐term decompensation. This suggests that while an LSM > 21 kPa is an imperfect predictor of a near‐term event, it remains an excellent marker of the underlying high‐risk state of established CSPH.

In our subgroup of patients with cACLD, both SSI‐2D‐LSM and VCTE‐LSM demonstrated good and comparable discriminative ability for predicting the first decompensation event. This finding reinforced the interchangeability of the two methods for clinical prognostication. In cACLD patients, the SSI‐2D‐LSM > 21 kPa cutoff also demonstrated high sensitivity for predicting one‐year decompensation, while specificity also improved. In clinical practice, the particularly high NPV of the SSI‐2D‐LSM > 21 kPa cut‐off indicates value to also identify those cACLD patients at negligible risk of short‐term decompensation.

However, our primary goal was to assess the prognostic value of SSI‐2D‐LSM for predicting decompensation events within the framework of the WFUMB ‘rule‐of‐4’ [29]. We therefore stratified patients into three clinically relevant groups based on these recommendations. First, we consolidated all patients with an LSM < 13 kPa into a single low‐risk category, representing non‐ACLD. Second, patients with an LSM of 13–21 kPa were combined into an intermediate group, indicative of ACLD. Finally, patients with an LSM > 21 kPa formed the high‐risk group, corresponding to a high probability of CSPH. Given that current guidelines recommend semiannual or annual LSM assessments for disease monitoring [11, 29, 39] we selected one‐year prediction of decompensation as a clinically relevant endpoint.

Our competing risk analysis further supports the prognostic power of the WFUMB ‘rule‐of‐4.’ In particular, patients with SSI‐2D‐LSM values above the high‐risk threshold had a markedly increased likelihood of developing decompensation within 1 year. The absence of events in the < 13 kPa group, however, required a methodological adjustment. We therefore dichotomised the cohort at the 21 kPa threshold (≤ 21 vs. > 21 kPa). This analysis confirmed that patients with an SSI‐2D‐LSM > 21 kPa have a significantly increased hazard for developing a decompensation event, highlighting the clinical utility of this cutoff for risk stratification.

These findings are consistent with and build upon existing literature. For instance, a study validating the VCTE‐based Baveno ‘rule‐of‐5’ similarly found no decompensation events in their ‘CSPH‐excluded’ group, while the ‘CSPH‐included’ group had the highest event rate [40]. Furthermore, our results parallel the work of Trebicka et al., who reported that patients with both an ARFI‐LSM ≥ 20 kPa and a MELD score ≥ 10 had a high one‐year decompensation risk of 26.6% [22].

A key strength of our approach lies in its simplicity for risk stratification in patients with cACLD. Whereas Trebicka et al. [22] incorporated MELD into their model to account for impaired liver function in a cohort including dACLD patients, only a minority of our cohort fell into this category. In the above mentioned study of Heilani et al. [34] combined models were developed using 2D‐SWE and PLT in analogy to VCTE‐based approaches, showing improved accuracy for predicting oesophageal varices and moderate accuracy for CSPH, with PLT adding value particularly in patients with intermediate stiffness values. In comparison, our findings indicate that a simple, stiffness‐based approach such as the ‘rule of four’ performs robustly for identifying CSPH, demonstrating good sensitivity and specificity at an SSI‐2D‐LSM threshold of > 21 kPa. Incorporating PLT improved diagnostic precision, particularly for ruling in CSPH, whereas lower stiffness cutoffs (≤ 13 kPa, especially when combined with normal PLT) effectively excluded CSPH with excellent sensitivity and NPV. Overall, these data support the clinical utility of SSI‐2D‐SWE alone as a pragmatic, non‐invasive rule for CSPH detection, while suggesting that combining LSM with PLT may further refine individualised risk stratification mirroring their synergistic role in the extended Baveno ‘Rule‐of‐5’ criteria [1].

Finally, our study suggests that the > 21 kPa SSI‐2D‐LSM cutoff has broad prognostic utility across different liver disease etiologies. This is a notable distinction from the VCTE‐based Baveno criteria, which are recommended primarily for patients with viral hepatitis, ALD, or non‐obese MASLD [1, 29]. Our cohort was etiologically diverse, including ALD, viral hepatitis and cholestatic/other liver disease. The consistent prognostic value of the SSI‐2D‐LSM cutoff in this mixed population supports its wider application, although future aetiology‐specific validation studies are certainly warranted.

Our study has limitations that should be acknowledged: First, the absence of decompensation events in the < 13 kPa group prevented a reliable estimation of subdistribution hazard ratios, which required us to dichotomise the cohort for the hazard analysis. Second, the smaller sample sizes in the lower‐risk groups (LSM < 13 kPa and 13–21 kPa) may introduce spectrum bias and limit the robustness of conclusions drawn specifically from these strata. Notably, this study was performed at a tertiary center and in a population with high prevalence of CSPH. The result presented here should not be uncritically generalised to other settings and populations with a lower pre‐test probability of CSPH. Third, the predominantly male cohort may limit the generalizability of our findings to female patients. Finally, as this was a single‐center study, our findings warrant external validation in different patient populations and healthcare settings.

Despite these limitations, our study possesses significant strengths. Its prospective design and the use of paired HVPG measurements, the gold standard for diagnosing CSPH, need to be acknowledged. The structured, prospective follow‐up for decompensation events further strengthens the validity of our prognostic findings. The decision to limit our primary endpoint to 1 year was also deliberate. While longer follow‐up would likely reveal more events, a one‐year timeframe aligns with clinical practice and the modern paradigm of dynamic risk stratification, where repeated LSM is used to regularly update a patient's prognosis [39].

In conclusion, this study validates the WFUMB ‘rule‐of‐4’ for SSI‐2D‐LSM as a robust tool for short‐term decompensation risk stratification in patients with ACLD. We confirmed that the > 21 kPa cutoff accurately identifies a high‐risk population, as validated by direct HVPG measurement. Patients in this group face a considerable one‐year decompensation risk. Given its strong prognostic value, its comparable performance to VCTE‐LSM and its practical integration into standard ultrasound systems, SSI‐2D‐LSM warrants broader implementation as an accessible, point‐of‐care tool for managing patients with advanced chronic liver disease.

## Author Contributions

All authors contributed either to study concept and design (C.S., T.R., D.J.M.B.) and/or data acquisition (all authors), analysis (C.S., T.R., D.J.M.B.) or interpretation (all authors). C.S., T.R. and D.J.M.B. drafted the manuscript, which was critically revised by all other authors. All authors read and approved the final manuscript.

## Disclosures and Conflicts of Interest

N.D. received travel support from Gilead. B.S.H. received travel support from Ipsen. L.B. received speaking fees from Chiesi, and Gilead. M.J. served as speaker and/or consultant for Gilead. B.Si. received travel support from AbbVie, Gilead, and Falk. M.T. received speaker fees from Falk Foundation, Gilead, Ipsen, Madrigal, Mirum; he advised for Agomab, Alfasigma, AssemblyBio, BoehringerIngelheim, Chemomab, Dexoligo Therapeutics, Falk Pharma, Gilead, Ipsen, Mirum, Pliant, ProQR Therapeutics, Rectify. He further received travel support from Falk Foundation and Gilead and research grants from Alnylam, Falk Pharma, Genentech, Gilead and UltraGenyx. He is also co‐inventor (service inventions as university employee) for patents on the medical use of NCA (filed by the Medical University of Graz). M.M. received grant support from Echosens, served as a speaker and/or consultant and/or advisory board member for AbbVie, AstraZeneca, Collective Acumen, Echosens, Eli Lilly, Gilead, Ipsen, Takeda, and W. L. Gore & Associates, and received travel support from AbbVie and Gilead. T.R. received grant support from Abbvie, Boehringer Ingelheim, Gilead, Intercept/Advanz Pharma, MSD, Myr Pharmaceuticals, Philips Healthcare, Pliant, Siemens and W. L. Gore & Associates; speaking/writing honoraria from Abbvie, Echosens, Gilead, GSK, Intercept/Advanz Pharma, Pfizer, Roche, MSD, Siemens, W. L. Gore & Associates; consulting/advisory board fee from Abbvie, Astra Zeneca, Bayer, Boehringer Ingelheim, Gilead, Intercept/Advanz Pharma, MSD, Resolution Therapeutics, Siemens; and travel support from Abbvie, Boehringer Ingelheim, Dr. Falk Pharma, Gilead, and Roche. D.J.M.B. received travel support from AbbVie, Siemens, and Gilead; and speaker fees from AbbVie and Röntgen Eisenstadt. The other authors declare no conflicts of interest.

## Supporting information


**AppendixS1:** liv70614‐sup‐0001‐AppendixS1.docx.
**Figure S1:** Cumulative Incidence of first decompensation stratified by SSI‐2D‐LSM‐LSM – considering liver transplantation and non‐liver‐related death as competing risks.
**Table S1:** Baseline characteristics of patients with HVPG measurements but without SSI‐2D‐LSM.
**Table S2:** Patient characteristics stratified by rule of four groups.

## Data Availability

The data that support the findings of this study are available from the corresponding author upon reasonable request.
