# Supplementary material for: Prognostication of Liver Disease Patients via the WFUMB ‘Rule‐of‐4’ Algorithm Using SSI‐2D‐SWE‐Based Liver Stiffness Measurement
Source: Liver Int. 2026 Apr 9;46(5):e70614. doi: 10.1111/liv.70614 (PMC13063207; doi:10.1111/liv.70614)

**Supplementary material**

**Supplementary tables**

**Table S-1: Baseline characteristics of patients with HVPG measurements but without SSI-2D-LSM**

|  | **HVPG only**  **(N=76)** | **SWE+ HVPG**  **(N=187)** | **p-value** |
| --- | --- | --- | --- |
| **Sex (W/M, %M)** | 47/29 (61.8%) | 59/128 (64.8%) | 0.376 |
| **BMI (kg/m²) [IQR]** | 25.8 [22.6 - 29.9] | 25.7 [22.9 - 28.8] | 0.979 |
| **VCTE LSM (kPa) [IQR]** | 28.7 [16.9 - 47.3] | 30.9 [16.6 - 59.1] | 0.774 |
| **MELD (points) [IQR]** | 11 [8 - 15] | 10 [9 - 15] | 0.600 |
| **Platelets (G/L) [IQR]** | 99 [74 - 158] | 117 [77 - 171] | 0.153 |
| **Thrombocytopenia <150 G/L (n, %)** | 55 (72.4%) | 122 (65.2%) | 0.331 |
| **HVPG (mmHg) [IQR]** | 13 [10 - 19] | 14 [9 - 20] | 0.871 |
| **CSPH (n, %) [IQR]** | 57 (75.0%) | 133 (71.1%) | 0.396 |
| **Ascites (n, %)** | 11 (14.5%) | 24 (12.8%) | 0.760 |
| **Follow-Up time (months) [IQR]** | 26.3 [17.3 - 29.5] | 24.1 [13.1 - 28.3] | 0.134 |
| **Decompensation rate at Y1 (n, %)** | 15 (19.7%) | 31 (16.6%) | 0.821 |

p-values comparing differences among groups

Abbreviations: W/M = Women/Men, BMI = Body mass index, IQR = Interquartile range, VCTE = Vibration-controlled transient elastography, LSM = Liver stiffness measurement, MELD = Model for End-stage Liver Disease, PLT = Platelets, G/L = 10⁹/L, HVPG = Hepatic venous pressure gradient, CSPH = Clinically significant portal hypertension

**Table S-2- Patient characteristics stratified by rule of four groups**

|  | **<5 (N=1)** | **5-9 (N=7)** | **9-13 (N=23)** | **13-17 (N=21)** | **17-21 (N=12)** | **>21 (N=123)** | p-value |
| --- | --- | --- | --- | --- | --- | --- | --- |
| **Age (years) [IQR]** | 40.0 [40.0 - 40.0] | 52.0 [41.0 - 57.5] | 59.0 [48.5 - 67.0] | 56.0 [52.0 - 62.0] | 59.5 [51.8 - 66.5] | 56.0 [50.5 - 64.0] | 0.449 |
| **Sex (W/M, %M)** | 1/0 (0%) | 2/5 (71.4%) | 7/16 (69.6%) | 6/15 (71.4%) | 4/8 (66.7%) | 39/84 (68.3%) | 0.804 |
| **BMI (kg/m²) [IQR]** | 23.8 [23.8 - 23.8] | 25.9 [23.7 - 28.9] | 25.4 [23.1 - 27.6] | 24.7 [22.6 - 29.0] | 23.7 [20.5 - 25.1] | 26.2 [23.3 - 29.7] | 0.384 |
| **Etiology:** |  |  |  |  |  |  |  |
| **ALD (n, %)** | 0 (0%) | 2 (28.6%) | 1 (4.3%) | 5 (23.8%) | 6 (50.0%) | 78 (63.4%) | **<0.001** |
| **MASLD (n, %)** | 0 (0%) | 0 (0%) | 2 (8.7%) | 5 (23.8%) | 2 (16.7%) | 10 (8.1%) |  |
| **Viral (n, %)** | 0 (0%) | 3 (42.9%) | 6 (26.1%) | 3 (14.3%) | 3 (25.0%) | 16 (13.0%) |  |
| **Other (n, %)** | 1 (100%) | 2 (28.6%) | 14 (60.9%) | 8 (38.1%) | 1 (8.3%) | 19 (15.4%) |  |
| **VCTE LSM (kPa) [IQR]** | 51.5 [51.5 - 51.5] | 6.60 [4.75 - 11.5] | 10.0 [7.05 - 14.6] | 18.6 [14.4 - 21.3] | 16.0 [12.3 - 20.1] | 48.3 [31.2 - 70.7] | **<0.001** |
| **Child Pugh Score (points) [IQR]** | 5 [5 - 5] | 5 [5 - 5] | 5 [5 - 5] | 5 [5 - 6] | 5 [5 - 6] | 7 [5 - 8] | **<0.001** |
| **MELD (points) [IQR]** | 6 [6 - 6] | 9 [7 - 10] | 9 [8 - 10] | 9 [7 - 10] | 8 [7 - 9] | 12 [10 - 16] | **<0.001** |
| **Platelets (G/L) [IQR]** | 153 [153 - 153] | 183 [115 - 205] | 181 [157 - 205] | 102 [83.0 - 171] | 110 [88.8 - 154] | 109 [69.0 - 153] | **<0.001** |
| **HVPG (mmHg) [IQR]** | 3 [3 - 3] | 6 [3 - 6] | 5 [3 - 7] | 11 [9 - 16] | 8 [7 - 11] | 17 [13 - 21] | **<0.001** |
| **CSPH (n, %) [IQR]** | 0 (0%) | 0 (0%) | 2 (8.7%) | 13 (61.9%) | 5 (41.7%) | 113 (91.9%) | **<0.001** |
| **Ascites (n, %)** | 0 (0%) | 0 (0%) | 0 (0%) | 0 (0%) | 1 (8.3%) | 23 (18.7%) | **<0.001** |
| **Any varices (n, %)** | 0 (0%) | 0 (0%) | 1 (4.3%) | 6 (28.6%) | 3 (25.0%) | 68 (55.3%) | **<0.001** |
| **High risk varices (n, %)** | 0 (0%) | 0 (0%) | 0 (0%) | 2 (9.5%) | 0 (0%) | 35 (28.5%) | **<0.001** |
| **SSI-2D-LSM (kPa) [IQR]** | 4.65 [4.65 - 4.65] | 6.20 [5.95 - 8.10] | 10.8 [9.75 - 11.5] | 14.3 [13.6 - 15.5] | 18.9 [17.8 - 20.1] | 58.4 [38.6 - 76.2] | **<0.001** |
| **Follow-Up time (months) [IQR]** | 16.4 [16.4 - 16.4] | 25.0 [13.6 - 27.3] | 21.7 [12.5 - 27.9] | 21.2 [15.6 - 27.4] | 18.3 [8.48 - 24.2] | 25.3 [13.1 - 28.5] | 0.579 |
| **Decompensation rate at Y1 (n, %)** | 0 (0%) | 0 (0%) | 0 (0%) | 2 (9.5%) | 1 (8.3%) | 28 (22.8%) | 0.053 |

p-values comparing differences among groups <0.05 bold

Abbreviations: W/M = Women/Men, BMI = Body mass index, IQR = Interquartile range, ALD = Alcohol-associated liver disease, MASLD = Metabolic dysfunction–associated steatotic liver disease, VCTE = Vibration-controlled transient elastography, LSM = Liver stiffness measurement, MELD = Model for End-stage Liver Disease, PLT = Platelets, G/L = 10⁹/L, HVPG = Hepatic venous pressure gradient, CSPH = Clinically significant portal hypertension

**Supplementary figures**

**Figure- S1: Cumulative Incidence of first decompensation stratified by SSI-2D-LSM-LSM – considering liver transplantation and non-liver-related death as competing risks**


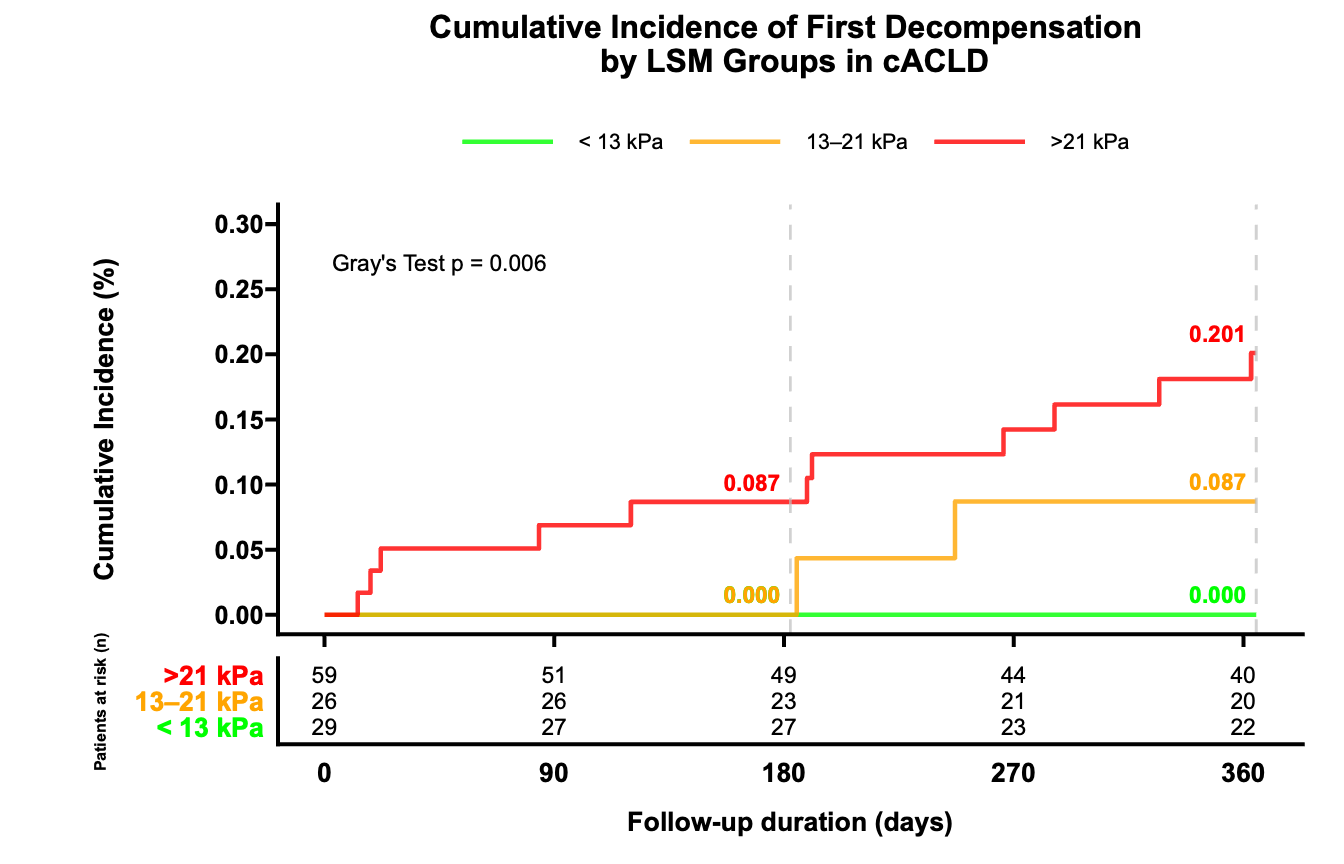

Supplement: Supplementary file 1 — AppendixS1: liv70614‐sup‐0001‐AppendixS1.docx. Figure S1: Cumulative Incidence of first decompensation stratified by SSI‐2D‐LSM‐LSM – considering liver transplantation and non‐liver‐related death as competing risks. Table S1: Baseline characteristics of patients with HVPG measurements but without SSI‐2D‐LSM. Table S2: Patient characteristics stratified by rule of four groups. [file LIV-46-0-s001.docx]
